# Cone‐Beam Computed Tomography Evaluation of Mineralized Tissue Formation Following Direct Pulp Capping With Calcium Silicate Materials

**DOI:** 10.1155/ijod/8162251

**Published:** 2026-10-27

**Authors:** Huynh-Anh Bui, Van-Khoa Pham

**Affiliations:** ^1^ Faculty of Dentistry University of Medicine and Pharmacy at Ho Chi Minh City Ho Chi Minh City Vietnam hcmut.edu.vn

**Keywords:** biodentine, calcium silicate biomaterials, cone-beam computed tomography (CBCT), direct pulp capping, mineral trioxide aggregate (MTA)

## Abstract

**Objectives:**

This study aimed to evaluate the efficacy of two calcium silicate biomaterials, mineral trioxide aggregate (MTA) and Biodentine (BD), as direct pulp capping (DPC) agents by assessing reparative mineralized tissue formation using cone‐beam computed tomography (CBCT).

**Materials and Methods:**

This randomized controlled, split‐mouth clinical trial involved 44 permanent premolars in patients aged 18–25 requiring orthodontic extraction. Teeth were randomly assigned for evaluation at 9–12 weeks and 13–16 weeks following DPC with either MTA or BD. Radiographic parameters, including dentin bridge thickness, continuity, and periapical status (CBCT periapical index [CBCT‐PAI]), were assessed in three dimensions. Bonferroni‐adjusted significance thresholds for multiple comparisons were *p*  < 0.0125 (dimensional) and *p*  < 0.025 (categorical).

**Results:**

All 44 teeth (100%) remained vital and asymptomatic, with no radiographic evidence of periapical lesions (CBCT‐PAI score 1), pulp necrosis, or inflammation. The BD group achieved a 100% incidence of dentin bridge formation at both follow‐up intervals, whereas the MTA group showed lower rates (63.6% at 9–12 weeks; 54.5% at 13–16 weeks). Even under strict Bonferroni correction, BD demonstrated significantly superior performance in dentin bridge thickness at 9–12 weeks (*p*  < 0.0125) and notably in both continuity and thickness at 13–16 weeks (*p*  < 0.025).

**Conclusion:**

Both MTA and BD are highly effective in maintaining clinical pulp vitality. However, BD exhibits significantly superior performance in promoting the consistent formation of thicker, more homogeneous, and continuous dentin bridges. CBCT was confirmed as an effective and precise modality for the early, detailed 3D assessment of mineralized tissue formation.

## 1. Introduction

The fundamental objective of direct pulp capping (DPC) is the preservation of dental pulp vitality. This therapeutic approach requires the application of a biocompatible material directly onto the exposed pulpal tissue, establishing a protective seal that shields the dentin‐pulp complex and stimulates the genesis of a mineralized barrier. Owing to the presence of odontoblasts, pulp fibroblasts, and mesenchymal stem cells within the dentin‐pulp complex, the dental pulp demonstrates a robust capacity for regeneration and modulation of inflammatory responses [1, 2]. Recent decades have witnessed substantial improvements in DPC clinical outcomes, largely driven by a deeper biological understanding of regenerative endodontics and the development of novel biomaterials.

For many years, calcium hydroxide was the gold standard for inducing mineralized hard tissue formation. Nevertheless, its application is hindered by notable limitations, such as gradual structural degradation, increased porosity, and the formation of tunnel defects over time. These structural weaknesses may permit bacterial microleakage, subsequently causing secondary pulpitis and compromising long‐term predictability [3]. To overcome these challenges, calcium silicate‐based cements have increasingly become the preferred alternative for vital pulp therapy since the 2000s. These materials are highly effective in managing pulpal trauma, inducing dentin bridge development, and enhancing overall pulpal survival [4].

When placed in direct contact with the pulp, hydrophilic tricalcium silicates foster a favorable healing milieu by modulating inflammatory cytokines [5]. Through the sustained release of calcium ions and calcium hydroxide, they trigger the proliferation and differentiation of key osteogenic and odontogenic cells, culminating in the construction of a reparative dentin barrier [5]. Among these, mineral trioxide aggregate (MTA) has been extensively utilized for pulpal injuries, consistently demonstrating high clinical success rates and the rapid stimulation of mineralized tissue [5–7]. Despite its efficacy, the widespread use of MTA is often limited by its prolonged setting time, challenging handling properties [4], and relatively high cost.

Recently, Biodentine (BD) has emerged as a highly bioactive alternative for dentin replacement. BD exhibits mechanical properties similar to dentin and possesses a high degree of purity, which reduces heavy elements and improves material properties [8]. Accumulating evidence suggests that BD is highly effective in accelerating the reparative dentinogenesis process during DPC procedures [7, 9, 10].

Radiographic imaging remains the primary method for assessing hard tissue neoformation postcapping. Although conventional two‐dimensional periapical radiographs are cost‐effective and readily accessible—frequently reporting 100% bridge formation success for both MTA and BD [11]—they inherently suffer from anatomical superimposition, failing to accurately depict the spatial volume and morphological quality of the reparative bridge [12].

In addition, cone‐beam computed tomography (CBCT) imaging allows for 3D reconstruction and precise measurement, eliminating superimposition noise from surrounding tissues [12, 13]. Previous CBCT evaluations have highlighted that while various materials can induce dentin bridging, the barriers formed by BD and MTA exhibit greater homogeneity and fewer structural defects compared to those induced by calcium hydroxide [12].

To comprehensively evaluate the dynamic processes of dentin‐pulp repair, we designed a randomized controlled clinical trial in accordance with CONSORT guidelines, integrating both clinical and advanced radiographic assessments. This study also evaluates the effectiveness of CBCT in detecting early periapical changes or mineralized tissue formation following DPC with BD compared to MTA over a 9–16‐week period posttreatment, thereby determining the efficacy of these calcium silicate materials in preserving the vitality of permanent teeth.

The null hypothesis tested was that there is no significant difference in the early formation, thickness, and continuity of mineralized tissue between MTA and BD following DPC.

## 2. Materials and Methods

### 2.1. Study Design

A split‐mouth, randomized controlled clinical trial was conducted to evaluate the comparative efficacy of two calcium silicate‐based capping agents. This robust design ensured that each participant received both MTA and BD on contralateral sides of the dental arch, thereby minimizing interindividual biological confounding factors.

The study question addressed whether there is any inferiority in mineralized tissue formation (via CBCT) after DPC between the two study groups using BD and MTA in permanent human teeth at two distinct evaluation periods: 9–12 weeks and 13–16 weeks postintervention with a sample size of 11 teeth per group.

Ethical approval for this research was obtained from the Ethics Council in Biomedical Research of the University of Medicine and Pharmacy at Ho Chi Minh City, Viet Nam, as per Approval Number 305/ĐHYD‐HĐĐĐ (21/05/2019). We registered the clinical trial with the retrospective Registration Number TCTR20191103001 on thaiclinicaltrials.org (03/11/2019).

Patients were provided with comprehensive information about the study’s objectives, methods, potential benefits, and any associated inconveniences. They provided signed informed consent and participated voluntarily. The research was conducted in a manner that respected the rights of patients. The pulp conservation treatment intervention using the DPC method on exposed pulp areas with MTA and BD materials was conducted in compliance with the licenses for these materials both globally and in Viet Nam.

### 2.2. Sample Size Determination

The sample size was calculated based on pooled data from previous comparative studies evaluating reparative dentin bridge formation following DPC with BD and MTA [11, 12]. By utilizing a split‐mouth design, interindividual biological variance is significantly reduced. An a priori power analysis determined that a minimum sample size of 11 pairs of teeth per observation cohort would provide statistical power (1−*β* = 0.08) of 80% at a 5% significance level (*α* = 0.05) to detect significant differences in the dimensional measurements of the mineralized tissue barriers.

### 2.3. Study Population

The study sample was selected from patients aged 18–25 presenting at the Department of Operative dentistry – Endodontics, Faculty of Dentistry, University of Medicine and Pharmacy at Ho Chi Minh City, Ho Chi Minh City. The enrollment screening was performed on patients necessitating the extraction of one or two pairs of permanent premolars (maxillary or mandibular) for orthodontic reasons. The inclusion and exclusion criteria are presented in Table 1. Clinical examination and X‐rays were performed at the baseline to assess the initial condition of the teeth and pulp. Structurally intact teeth with no caries or minor enamel damage were selected based on the International Caries Detection and Assessment System (ICDAS‐II) scale with scores 0–2 [14], and the periodontal pocket depth was assessed based on the Classification of Periodontal and Peri‐Implant Diseases and Conditions [15]. There was a requirement for no clinical or radiographic symptoms of irreversible pulpitis (according to Endodontic Diagnosis, AAE Consensus Conference Recommended Diagnostic Terminology, 2013) [16] and positive responses to thermal, electrical pulp tests, and percussion tests consistent with normal dental pulp vitality.

**Table 1:** Inclusion and exclusion criteria.

Inclusion criteria	Exclusion criteria
1. Healthy patients aged 18–25 years 2. Requiring extraction of 1–2 permanent premolar pairs for orthodontics 3. Symmetrical tooth pairs (shape, position, and orientation) 4. Intact structures teeth with no caries or minor enamel damage (ICDAS‐II scores 0–2) 5. No periodontal pockets deeper than 4 mm 6. No clinical symptoms of reversible pulpitis 7. Positive response to thermal/electrical tests and the percussion test 8. No periapical changes on radiographs 9. Radiographic evidence of completed apical closure 10. No history of dental trauma	1. Immunocompromised systemic diseases 2. Long‐term use of antibiotics or antiinflammatory drugs 3. History of allergy to local anesthetics or their components 4. Large cavities or large fillings close to the pulp 5. Excessive tooth mobility or periodontal disease, presence of fistulas or swelling 6. Clinical symptoms of irreversible pulpitis 7. Negative response to thermal/electrical tests and tenderness to percussion. 8. Radiographic evidence of periapical radiolucencies, internal resorption, or pulp calcification 9. Pregnancy (requirements for radiographs) 10. Uncontrollable pulpal bleeding upon exposure

### 2.4. Sampling Method

A convenience sample was drawn from orthodontic patient records using a systematic random sampling method (an interval of 2). The first subject invited to participate was randomly selected from the eligible records. Subsequently, every second eligible record was invited. If a patient declined, the next eligible record was invited.

A split‐mouth design with two independent cohorts was established based on the time of extraction: “9–12 weeks” or “13–16 weeks.” A total of 74 permanent premolars (37 pairs of symmetrical teeth) from 23 orthodontic patients were initially screened for study eligibility. Following the exclusion criteria, 56 permanent premolars (28 pairs of teeth) from 15 patients were selected. They were informed about the study’s objectives, methods, potential benefits, and risks and provided with written informed consent. Each patient in a cohort received either MTA Angelus (Angelus Solucoes Odontologicas, Londrina, Brazil) or BD (Septodont, France) as the pulp capping material.

For the split‐mouth allocation, the specific side of the dental arch receiving either MTA or BD was assigned using a randomization process based on computer‐generated random numbers (and a coin toss for side allocation). To ensure strict allocation concealment, the treatment assignments were sealed in sequentially numbered opaque envelopes, which were opened by the dental assistant only at the time of the DPC procedure. Although the operating clinician could not be blinded during the intervention due to the distinct physical and handling characteristics of MTA and BD, a rigorous blinding protocol was implemented for the outcome evaluation. Specifically, the radiologist who performed the CBCT measurements and categorical assessments was completely blinded to the treatment allocation and the specific capping material applied to each tooth.

The outcomes were evaluated at two post‐DPC intervals: after 9–12 weeks (group pair MTA1‐BD1) or after 13–16 weeks (group pair MTA2‐BD2).

Throughout the study, 30 teeth were excluded due to participant withdrawal, relocation, extraction delays, and extraction complications. Ultimately, a total of 22 pairs (44 teeth) in this split‐mouth design successfully completed the entire study protocol and met clinical and radiographic criteria for the final analysis (Figure 1).

**Figure 1:**
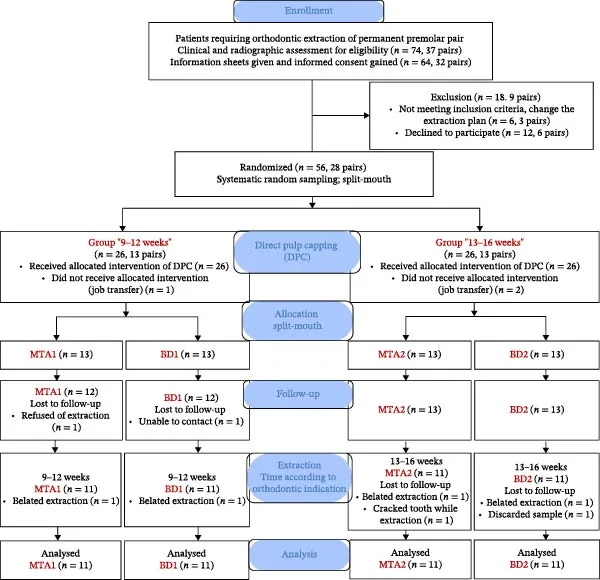
Consort flow diagram.

### 2.5. The Research Process Was Conducted in Two Phases as Follows

#### 2.5.1. Phase 1: Baseline Clinical Examination

Baseline evaluations involved a comprehensive recording of subjective symptoms, physical signs, and radiographic findings. Initially, we conducted a clinical examination, inquiring about and recording subjective symptoms such as discomfort, sensitivity, and pain [17].

Prior to index recording, all patients underwent supragingival scaling and polishing.

The following clinical indices were recorded:

• Caries status: Classified according to the ICDAS‐II score [14],
• Periodontal status: Plaque index (PI), gingival index (GI) [18], and periodontal disease classification index [15],
• Tooth mobility: Evaluated using the Miller mobility index.

Pulp vitality and sensitivity tests were performed in triplicate on the target teeth as well as on contralateral or adjacent healthy teeth to serve as controls.

• Pulp testing was conducted using an electric pulp tester, with the probe placed on the middle third of the facial surface. A response score between 1 and 6 was considered normal pulpal responses.
• Percussion testing was performed with moderate force using the end of a mirror handle in a vertical direction on the occlusal, facial, and lingual surfaces of the tooth. Patients were asked to report any discomfort, tenderness, or sharp pain. Positive vertical percussion was utilized as a clinical indicator of potential periapical inflammation associated with advanced pulpitis or pulpal necrosis.

The baseline tooth shade was documented via clinical photography using the Vita Toothguide 3D Master shade guide (VITA Zahnfabrik, Bad Säckingen, Germany). The CBCT scans were obtained for all subjects prior to the DPC procedure to establish a radiographic reference for periapical health and dentin tissue formation.

#### 2.5.2. Phase 2: DPC Procedures

##### 2.5.2.1. Clinical Procedure

Following the administration of local anesthesia using 1.8 mL of 2% lidocaine with 1:100,000 epinephrine (Septodont, Saint‐Maur‐des‐Fossés, France), absolute isolation was achieved using a rubber dam. The tooth was cleaned with a prophylactic brush, and the surface was disinfected with 2% chlorhexidine (CHX). Standardized cavities (measuring ~2 × 2 mm with a depth of 3–4 mm) were mechanically prepared using a sterile high‐speed carbide round bur under copious saline irrigation and cleansed with 1.5% sodium hypochlorite (NaOCl) (Hyposol, Prevest Denpro, Chile). Pulpal hemostasis was secured via the application of sterile 0.9% saline‐soaked cotton pellets with gentle pressure for 3–8 min.

Once bleeding was controlled, the exposure site was disinfected with 3% NaOCl prior to the placement of the capping agents. Patients exhibiting uncontrollable pulpal hemorrhage were systematically excluded from the study. The pulp capping protocol was established in accordance with the European Society of Endodontology (ESE) guidelines about the management of deep caries and the exposed pulp [19].

In the MTA group, we separated into 2 groups, MTA1 (9–12 weeks) and MTA2 (13–16 weeks); each cohort received MTA over the pulp exposure. MTA was mixed and placed as a 1.5–2 mm‐thick layer covering the exposed pulp and extending 0.5–1 mm beyond the exposure. A thin moist cotton pellet was placed over the MTA layer and temporarily filled with glass ionomer cement (GIC Fuji IX, GC, Tokyo, Japan) to provide an immediate temporary coronal seal.

In the BD group, similarly divided into BD1 (9–12 weeks) and BD2 (13–16 weeks), BD was mixed according to the manufacturer’s instructions, then placed to create a similar layer thickness over the exposure, and similarly filled with GIC Fuji IX.

##### 2.5.2.2. Follow‐Up

One week postoperatively, the GIC was completely replaced by the G‐aenial composite (GC, Tokyo, Japan). Clinical examinations and pulp vitality tests were repeated, along with the taking of photographs and radiographs.

The researcher provided a questionnaire, and the patient recorded subjective sensations on a visual analog scale (VAS) using the Wong–Baker faces scale with increasing levels of discomfort or pain corresponding to scores from 0 to 10 [20].

Patients were advised on dentin hypersensitivity management and, when necessary, prescribed analgesics and antibiotics. Percussion tests and electric pulp tests were conducted to assess pulp sensitivity. Clinical symptoms, including swelling, abscess formation, fistula, tooth mobility, and periodontal status, were recorded.

Treatment success was defined as the absence of clinical signs of pulpal or periapical disease, normal response to pulp vitality tests, and absence of significant pain symptoms with the percussion test or during mastication.

Subsequent assessment intervals were scheduled at 4–8, 9–12, and 13–16 weeks with the same evaluation procedures. The tooth extraction was performed after 9–16 weeks posttreatment. Prior to extraction, clinical reevaluations, pulp vitality tests were repeated, accompanied by postoperative radiographs and subjective assessments via questionnaires. Atraumatic extraction was then performed by an oral surgeon under local anesthesia. Patients were instructed on postoperative care, prescribed analgesics, and scheduled for postextraction follow‐up.

##### 2.5.2.3. Radiographic Evaluation

CBCT scans were performed at baseline, during follow‐up visits, and postextraction.

CBCT scans were acquired using a Planmeca Promax 3D classic digital imaging system (Planmeca Oy, Helsinki, Finland). The CBCT parameters were set to 90–120 kVp and 14 mA using a 3D complementary metal‐oxide‐semiconductor (CMOS) sensor. The endodontic imaging mode provided high‐resolution slices with a voxel size of 0.075 mm, field of view (FOV) of 5 × 5 cm, slice size of 1024 × 1024 pixels, and scan time of 8 s. This specialized mode allowed for a small, localized FOV, focusing specifically on only the study teeth.

Additionally, safety precautions were implemented during radiographic procedures in compliance with the ALARA principle, and all patients were required to wear lead aprons covering the thyroid and submandibular region. Scans were scheduled 2–8 weeks apart to minimize radiation accumulation and facilitate metabolic clearance. Furthermore, the dental periapical X‐ray dose for the digital film was maintained at very low levels of effective dose [21], ranging from 2 to 9 μSv per instance, while the CBCT film effective dose was ~19–44 μSv per instance [22].

Three‐dimensional digital images were analyzed using Planmeca Romexis software. The images were normalized perpendicular to the long axis of the tooth as much as possible in three planes: axial, coronal, and sagittal, and measurements were then taken on the corresponding slices at the cementoenamel junction, 0.5 mm apical to the cementoenamel junction, and 0.5 mm coronal to the cementoenamel junction.

The radiographic recorded parameters included the following:

• Dentin bridge thickness following treatment (at T2 and T3) was measured on the coronal, sagittal, and axial planes using a four‐level scale: (1) ≥0.25 mm; (2) 0.1–0.24 mm; (3) 0.01–0.09 mm; (4) and no dentin bridge.
• Dentin bridge continuity was assessed by observing the continuity or discontinuity of the dentin barrier formation or dentin bridge border on three planes relative to the root dentin surrounding the exposed pulp using a four‐level scale: (1) Complete dentin bridge covering the exposed pulp; (2) partial or incomplete dentin bridge, covering more than half of the exposed pulp but not completely; (3) initial dentin bridge covering less than half of the exposed pulp or distant from the exposed pulp; and (4) no dentin bridge.
• Presence of periapical lesions: Assessed using the CBCT periapical index (CBCT‐PAI) [23].

Radiographic treatment success was defined as the presence of new mineralized tissue, the absence of ligament widening or periapical radiolucency, and no evidence of internal or external resorption. All measurements were performed independently three times, 1 week apart, by a radiologist. Intraobserver reliability was assessed using the intraclass correlation coefficient (ICC). The ICC was ≥0.93 (95% CI: 0.77–0.98), and the calculated Cohen’s kappa coefficient was 0.84, indicating high reliability.

This study’s meticulous design and stringent protocols aimed to rigorously evaluate the effectiveness of DPC using MTA and BD, with the findings anticipated to contribute valuable insights into minimally invasive endodontic therapies.

### 2.6. Statistical Analysis

Data analysis was conducted using SPSS 20.0 statistical software (IBM Corp., Armonk, NY, USA) with a 95% confidence level. Conclusions were based on the *p*‐value, with a *p*‐value ≤ 0.05 indicates a statistically significant difference.

Mann–Whitney *U*/Wilcoxon signed‐rank tests were used to compare continuous variables, specifically the quantitative measurements of dentin bridge thickness. Fisher’s exact test was applied to analyze categorical data, including the four‐level scale scores for dentin bridge thickness and continuity.

To control the inflation of Type I error rates associated with evaluating multiple outcome measures within the same cohort, a Bonferroni correction was retrospectively applied. For the multiple comparisons of the four continuous dimensional parameters (vestibular–lingual, mesial–distal, apical–coronal, and mean thickness) of the dentin bridge, the significance threshold was adjusted to *p*  < 0.0125 (*α* = 0.05/4). For the two categorical four‐level scale assessments (thickness score and continuity score), the significance threshold was adjusted to *p*  < 0.025 (*α* = 0.05/2).

## 3. Result

### 3.1. Periapical Lesions and Internal and External Resorption Status

No clinical signs of pulpitis were observed regarding pain, swelling, and sinus tracts; pulp vitality was confirmed via electric pulp testing and percussion testing. A thorough radiographic examination of all teeth in this study revealed no evidence of widened periodontal ligaments or discontinuities in the lamina dura. No periapical lesions and no internal or external resorption signs were found on CBCT, corresponding to score 1 (no lesion) in the CBCT‐PAI.

### 3.2. Dentin Bridge Evaluation

#### 3.2.1. Group Pair MTA1‐BD1 (9–12 Weeks)

Regarding the mean dentin bridge thickness measurements, the BD1 group demonstrated significantly greater dentin bridge thickness in several key continuous dimensions, notably the vestibular–lingual, apical–coronal, and overall mean thickness at the preextraction interval (T2.1) following the Bonferroni correction (significance threshold was adjusted to *p*  < 0.0125) (Table 2).

**Table 2:** Mean dentin bridge thickness on CBCT in group pair MTA1‐BD1.

Radiographic parameters	Time points	MTA1 (*n* = 11) Mean ± SD (mm)	BD1 (*n* = 11) Mean ± SD (mm)	*p*‐Value
Dentin bridge thickness (vestibular–lingual)	T2.1	0.47 ± 0.59^Aa^	1.37 ± 0.55^Ba^	**0.003**
	T3.1	0.96 ± 0.93^Ab^	1.71 ± 0.66^Ab^	0.045
Dentin bridge thickness (mesial–distal)	T2.1	0.29 ± 0.37^Aa^	0.62 ± 0.28^Aa^	0.077
	T3.1	0.50 ± 0.58^Ab^	0.89 ± 0.36^Ab^	0.047
Dentin bridge thickness (apical–coronal)	T2.1	0.20 ± 0.24^Aa^	0.79 ± 0.65^Ba^	**0.001**
	T3.1	0.37 ± 0.35^Ab^	1.19 ± 0.88^Bb^	0.006
Mean dentin bridge thickness	T2.1	0.32 ± 0.39^Aa^	0.93 ± 0.44^Ba^	**0.003**
	T3.1	0.61 ± 0.58^Ab^	1.26 ± 0.54^Ab^	0.019

*Note: p*‐Values were calculated using the Mann–Whitney *U*/Wilcoxon signed‐rank test, adjusted significance threshold: *p*  < 0.0125. Different uppercase superscript letters (A, B) within the same row indicate statistically significant intergroup differences at the same time point. Different lowercase superscript letters (a, b) within the same column indicate statistically significant intragroup differences across different observation periods. T2.1: CBCT time taken at 9–12 weeks of DPC placement, before tooth extraction (MTA1‐BD1), T3.1: CBCT time taken w ithin 48 h after tooth extraction (MTA1‐BD1). Bold values in *p*‐value column denote statistically significant differences (*p* < 0.0125 for continuous dimensional parameters and *p* < 0.025 for categorical scores following Bonferroni correction for multiple comparisons).

In the MTA1 group, 7/11 teeth (63.6%) exhibited a dentin bridge ≥ 0.25 mm, while 4/11 teeth (36.4%) had no dentin bridge present. The BD1 group showed consistent bridge formation, with 10/11 teeth (90.9%) having a dentin bridge ≥ 0.25 mm, 1/11 teeth (9.1%) having a dentin bridge 0.1–0.24 mm, and no instances of absent bridge formation (Table 3). Statistical results showed no significant difference between the two groups, MTA1 and BD1, in the same pair in dentin bridge thickness in a four‐level scale (Fisher’s exact test, *p*  > 0.025). The dentin bridge continuity scores showed a strong trend favoring BD but fell just outside the highly conservative adjusted significance thresholds *p* = 0.025 (Table 3).

**Table 3:** Four‐level scale assessment of dentin bridge on CBCT in group pair MTA1‐BD1.

Radiographic parameters	Time points	MTA1 (*n* = 11) Score *n* (%)				BD1 (*n* = 11) Score *n* (%)				*p*‐Value
		(1)	(2)	(3)	(4)	(1)	(2)	(3)	(4)	
Dentin bridge thickness	T2.1	5 (45.5)	0	0	6 (54.5)^Aa^	10 (90.9)	0	0	1 (9.1)^Aa^	0.063
	T3.1	7 (63.6)	0	0	4 (36.4)^Aa^	10 (90.9)	1 (9.1)	0	0^Aa^	0.090
Dentin bridge continuity	T2.1	3 (27.3)	2 (18.2)	0	6 (54.5)^Aa^	7 (63.6)	1 (9.1)	2 (18.2)	1 (9.1)^Aa^	0.046
	T3.1	3 (27.3)	2 (18.2)	2 (18.2)	4 (36.4)^Aa^	9 (81.8)	1 (9.1)	1 (9.1)	0^Aa^	0.026

*Note: p*‐Values were calculated using Fisher’s exact test, adjusted significance threshold: *p*  < 0.025. Different uppercase superscript letters (A, B) within the same row indicate statistically significant intergroup differences between the capping materials. T2.1: CBCT time taken at 9–12 weeks of DPC placement, before tooth extraction (MTA1‐BD1), T3.1: CBCT time taken w ithin 48 h after tooth extraction (MTA1‐BD1).

#### 3.2.2. Group Pair MTA2‐BD2 (13–16 Weeks)

Regarding the mean dentin bridge thickness measurements, the mesial–distal and apical–coronal thicknesses were significantly larger in the BD2 group. However, overall mean dentin thickness showed no significant difference at the preextraction interval following the Bonferroni correction, as the *p*‐value exceeded the adjusted significance threshold of *p*  < 0.0125 (Table 4).

**Table 4:** Dentin bridge thickness on CBCT in group pair MTA2‐BD2.

Radiographic parameters	Time points	MTA2 (*n* = 11) Mean ± SD (mm)	BD2 (*n* = 11) Mean ± SD (mm)	*p*‐Value
Dentin bridge thickness (vestibular–lingual)	T2.2	0.60 ± 0.87^Aa^	1.38 ± 0.57^Aa^	0.023
	T3.2	0.74 ± 0.94^Ab^	1.66 ± 0.57^Ab^	0.110
Dentin bridge thickness (mesial–distal)	T2.2	0.28 ± 0.43^Aa^	0.85 ± 0.26^Ba^	**0.006**
	T3.2	0.40 ± 0.57^Ab^	1.07 ± 0.32^Bb^	**0.007**
Dentin bridge thickness (apical–coronal)	T2.2	0.21 ± 0.30^Aa^	0.83 ± 0.76^Ba^	**0.003**
	T3.2	0.31 ± 0.38^Ab^	0.94 ± 0.80^Ab^	0.025
Mean dentin bridge thickness	T2.2	0.37 ± 0.51^Aa^	1.02 ± 0.45^Aa^	0.210
	T3.2	0.48 ± 0.59^Ab^	1.22 ± 0.46^Ab^	0.190

*Note: p*‐Values were calculated using the Mann–Whitney *U*/Wilcoxon signed‐rank test, adjusted significance threshold: *p*  < 0.0125. Different uppercase superscript letters (A, B) within the same row indicate statistically significant intergroup differences at the same time point. Different lowercase superscript letters (a, b) within the same column indicate statistically significant intragroup differences across different observation periods. T2.2: CBCT time taken at 13–16 weeks of DPC placement, before tooth extraction (MTA2‐BD2), T3.2: CBCT time taken within 48 h after tooth extraction (MTA2‐BD2). Bold values in *p*‐value column denote statistically significant differences (*p* < 0.0125 for continuous dimensional parameters and *p* < 0.025 for categorical scores following Bonferroni correction for multiple comparisons).

In the MTA2 group, 5/11 teeth (45.5%) had a dentin bridge ≥ 0.25 mm, 1 tooth (9.1%) had a size of 0.01–0.09 mm, and 5/11 teeth (45.5%) lacked a dentin bridge. The BD2 group, however, achieved a 100% incidence (11/11 teeth) of dentin bridge ≥ 0.25 mm and no tooth without a dentin bridge (Table 5). The categorical scores for both dentin bridge thickness (*p* = 0.004 at T2.2; *p* = 0.012 at T3.2) and continuity (*p* = 0.006 at T2.2; *p* = 0.004 at T3.2) were highly significant (Table 5). BD demonstrated statistically superior performance over MTA in a four‐level scale assessment of dentin bridge thickness and the continuity of the dentin bridge, with a highly significant value (*p*  < 0.025) even after the strict Bonferroni correction (Table 5).

**Table 5:** Four‐level scale assessment of dentin bridge in group pair MTA2‐BD2.

Radiographic parameters	Time points	MTA2 (*n* = 11) Score *n* (%)				BD2 (*n* = 11) Score *n* (%)				*p*‐Value
		(1)	(2)	(3)	(4)	(1)	(2)	(3)	(4)	
Dentin bridge thickness	T2.2	4 (36.4)	0	0	7 (63.6)^Aa^	11 (100)	0	0	0^Ba^	**0.004**
	T3.2	5 (45.5)	0	1 (9.1)	5 (45.5)^Aa^	11 (100)	0	0	0^Ba^	**0.012**
Dentin bridge continuity	T2.2	3 (27.3)	1 (9.1)	0	7 (63.6)^Aa^	6 (54.5)	2 (18.2)	3 (27.3)	0^Ba^	**0.006**
	T3.2	4 (36.4)	1 (9.1)	1 (9.1)	5 (45.5)^Aa^	11 (100)	0	0	0^Ba^	**0.004**

*Note: p*‐Values were calculated using Fisher’s exact test, adjusted significance threshold: *p*  < 0.025. Different uppercase superscript letters (A, B) within the same row indicate statistically significant intergroup differences between the capping materials. T2.2: CBCT time taken at 13–16 weeks of DPC placement, before tooth extraction (MTA2‐BD2), T3.2: CBCT time taken within 48 h after tooth extraction (MTA2‐BD2). Bold values in *p*‐value column denote statistically significant differences (*p* < 0.0125 for continuous dimensional parameters and *p* < 0.025 for categorical scores following Bonferroni correction for multiple comparisons).

Figure 2 illustrates the distribution of the mean dentin bridge thickness across groups. BD consistently exhibited higher median thickness values than MTA at both 9–12 and 13–16 weeks. While BD data showed a more concentrated distribution, MTA groups presented a wider range, including several cases with zero thickness. Notably, measurements significantly increased from the preextraction (intraoral) phase (T2) to the postextraction (extraoral) phase (T3) for all groups.

**Figure 2:**
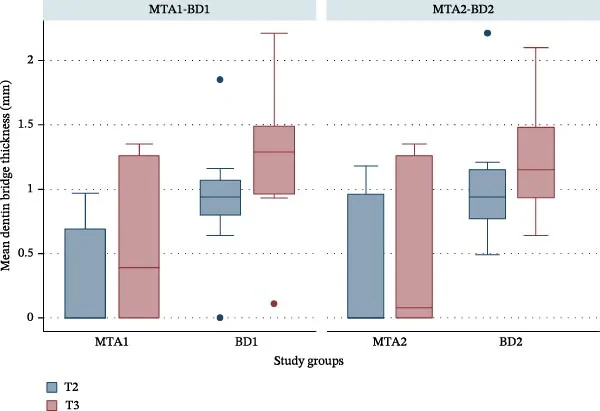
Distribution of mean dentin bridge thickness (mm) on CBCT images across the study groups at 9–12 weeks and 13–16 weeks observation periods.

### 3.3. Morphological Variations of Dentin Bridges on CBCT

Morphologically, the majority of cases exhibited a band of mineralized tissue that completely or partially covered the exposed pulp in both MTA and BD groups. In three dimensions, the continuous dentin bridge was located close to or 0.3–0.5 mm apical to the exposure site, exhibiting a radiopacity level comparable to the surrounding natural dentin (Figures 3 and 4). We observed one case exhibiting diffuse mineralization within the pulp chamber in the BD1 group, characterized by the appearance of a remarkably large radiopaque mass (Figure 5). Additionally, we observed two cases (one in each group) where the dentin bridge formed distant from the exposure site, presenting a distinct radiopaque connecting branch extending from the dentin bridge toward the center of the exposed pulp area (Figure 6).

**Figure 3:**
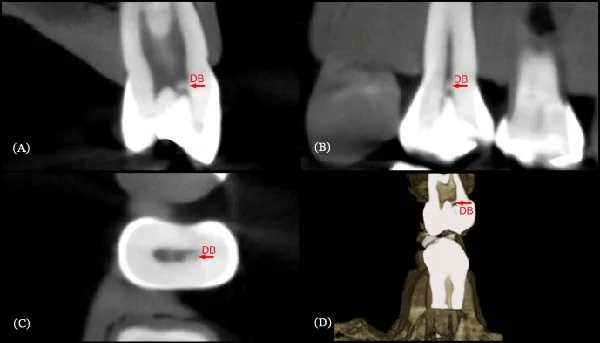
Continuous dentin bridges formed after direct pulp capping in an upper premolar (MTA1 group). Red arrows (DB‐dentin bridge) indicate the mineralized tissue across the (A) coronal, (B) sagittal, and (C) axial planes, alongside (D) a 3D volumetric reconstruction.

**Figure 4:**
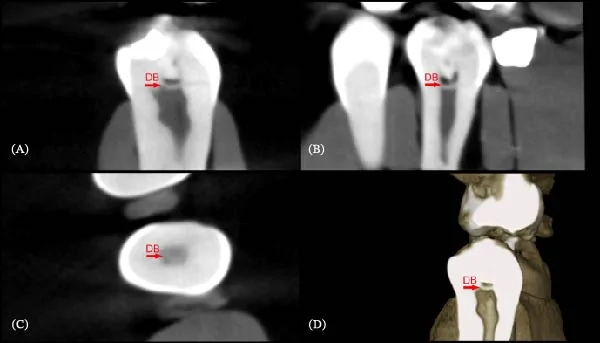
Continuous dentin bridges formed after direct pulp capping in a lower premolar (BD2 group). Image layout and annotations (A–D, DB) are identical to Figure 3.

**Figure 5:**
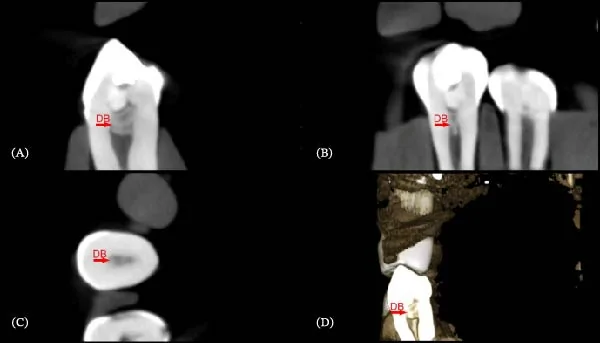
Diffuse mineralization within the pulp chamber formed after direct pulp capping in a lower premolar (BD1 group). Image layout and annotations (A–D, DB) are identical to Figure 3.

**Figure 6:**
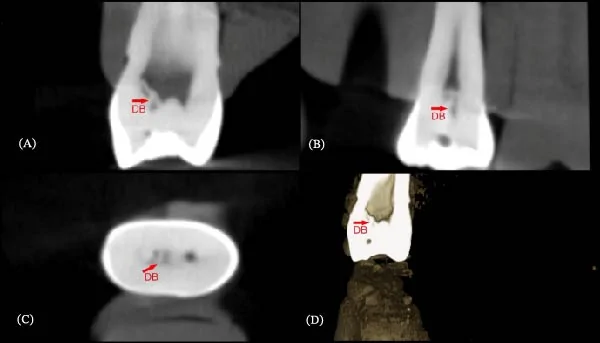
A radiopaque branch connecting the exposed pulp area to a distant dentin bridge formed after direct pulp capping in the upper premolar (BD2 group). Image layout and annotations (A–D, DB) are identical to Figure 3.

## 4. Discussion

The fundamental objective of DPC is to preserve pulpal vitality by establishing a reliable seal over the exposed area and promoting the development of a reparative mineralized hard tissue barrier. In this context, our investigation utilized CBCT to conduct a detailed 3D volumetric and morphological analysis of dentin bridge genesis, thereby facilitating a more comprehensive analysis of calcium silicate‐based materials.

Regarding clinical outcomes, although the BD group exhibited a trend toward enhanced dentin bridge formation, both capping agents yielded an excellent 100% clinical success rate. Throughout the observation period, all treated premolars preserved normal pulp vitality, and there was no radiographic indication of root resorption or periapical pathosis. These favorable outcomes corroborate the results of Katge and Patil [11], who also documented a complete absence of periapical disease in radiographic assessments and a 100% success rate for both MTA and BD. This exceptionally high success rate is noteworthy when compared to the findings by Lipski, who noted an 82.6% success rate [24] and the findings by Parinyaprom, who noted a failure rate of 2/27 teeth in the MTA group due to pulpitis pain, while 1/28 teeth in the BD group exhibited negative pulp test results [25]. The optimal outcomes in our study may be attributed to the strict inclusion of structurally intact premolars scheduled for orthodontic extraction, ensuring a baseline of healthy pulp free from prior inflammation. Notably, all treated teeth maintained vitality throughout the study, regardless of the radiographic presence or absence of dentin bridge formation. Interestingly, the absence of a distinct dentin bridge on CBCT was observed much more frequently in the MTA groups (10/22 teeth) compared to the BD groups (1/22 teeth). This challenges traditional assertions by some authors [25, 26] who maintain that a continuous dentin bridge is a strict prerequisite for successful treatment. We propose that macroscopic dentin bridging may not be the sole protective mechanism. Reactive dentin deposition along the pulpal walls, which reduces dentin permeability, may also contribute significantly to preserving pulp vitality, particularly in those MTA cases where reparative barriers are not radiographically evident. Furthermore, the nonhomogeneous structure of some dentin bridges implies that a hermetic seal of the pulp from the oral environment may not always be achieved, leaving a theoretical risk for bacterial penetration. Therefore, questions remain regarding the optimal dentin phenotype for pulp healing and vitality. Nevertheless, these findings highlight the diversity of pulpal responses—extending beyond macrostructural dentin bridge formation—and confirm the remarkable efficacy of calcium silicate‐based materials, offering clinicians a sound evidence‐based rationale for their application in pulp preservation therapies.

Transitioning to the radiographic outcomes, the null hypothesis—which posited that there would be no significant differences in the early formation, thickness, and continuity of mineralized tissue between MTA and BD—was rejected. Our 3D volumetric evaluations revealed that BD demonstrated significantly superior performance in promoting reparative dentinogenesis. By combining levels (1), (2), and (3) of the dentin bridge thickness, BD groups demonstrated significantly superior performance in promoting mineralized tissue formation, and they exhibited a 100% incidence rate (11/11 teeth) postextraction at both follow‐up intervals (T3.1, Table 3 and T3.2, Table 5). In the MTA total group, dentin bridge images were just found in 63.6% (7/11 teeth) at 9–12 weeks (T3.1, Table 3) and 54.5% (6/11 teeth) at 13–16 weeks (T3.2, Table 5). Furthermore, BD consistently yielded bridges that were significantly thicker and more continuous than those formed by MTA, a finding that remained statistically robust even after applying conservative Bonferroni adjustments.

This significant disparity in dentin bridge quality can be fundamentally explained by the compositional and physicochemical differences between the two materials. The mechanism of reparative dentinogenesis is primarily driven by the hydration of calcium silicates, leading to the release of calcium and hydroxide ions. This alkaline environment modulates inflammatory cytokines and stimulates the recruitment, proliferation, and differentiation of pulp stem cells into odontoblast‐like cells. BD is formulated with highly pure tricalcium silicate and utilizes zirconium oxide as a radiopacifier, deliberately excluding heavy metals such as bismuth oxide found in MTA. Furthermore, its considerably smaller particle size and the incorporation of calcium chloride as a setting accelerator allow BD to exhibit a more rapid, voluminous, and sustained release of calcium ions compared to MTA. This accelerated and intense bioactivity initiates an earlier healing cascade, which translates clinically into the faster formation of the thicker, denser, and more structurally homogeneous dentin bridges observed in our BD cohort. It is also important to note that the observed “decrease” in the incidence of dentin bridges in the MTA group from 9 to 12 weeks to 13–16 weeks is not due to the longitudinal resorption or disappearance of the mineralized tissue. Since this study utilized a cross‐sectional cohort design based on the time of extraction with different teeth at each interval, this difference merely reflects intersample biological variability within the distinct cohorts, rather than material failure over time. The overall incidence of dentin bridges in our study is slightly lower than that reported by Muruganandhan, who observed 100% formation for both materials [27], but higher than the 6‐week evaluation by Nowicka et al. [12]. Although CBCT scans showed significant advantages in detecting early signs of dentin mineralization, Nowicka’s study had a shorter study period (6 weeks) [12] compared to this study (9–16 weeks), which may have caused the difference in the number of teeth without a dentin bridge on CBCT. This indicates that the timeframe of 9–16 weeks is a critical window for observing ongoing mineralization.

The continuity of dentin bridges was significantly higher in the BD groups compared to the MTA group at both periods (Tables 3 and 5). Some cases exhibited incomplete bridges or mineralized tissue situated distant from the exposure pulp site. Compared to Nowicka’s findings [10] with 6/11 teeth in the MTA group and 7/11 teeth in the BD group, our results showed a significant difference between the two groups at two periods. However, due to the maximum follow‐up time of 16 weeks, the continuity of dentin bridges had a different ratio compared to the long‐term 36‐month evaluation by Peskersoy that showed similar high continuity for both materials (86.7% for MTA and 85.7% for BD) [28]. BD’s superior early performance in our study is likely linked to its high purity and improved mechanical properties, allowing for a faster release of calcium ions to initiate the healing process.

We observed an increase in mean dentin thickness (vestibular–lingual, mesial–distal, and apical–coronal) between the two CBCT imaging time points, T2.1–T3.1 and T2.2–T3.2 (Tables 2 and 4), in both MTA and BD groups. The use of CBCT confirmed a discernible difference in measurements between the preextraction (intraoral, T2) and postextraction (extraoral, T3) phases. This highlights the inherent limitations of CBCT imaging in assessing teeth within the oral cavity environment, where visualization and exact measurements may be slightly compromised by interference from the surrounding anatomical structures [13].

Finally, regarding the morphological variations observed in our CBCT scans, the typical presentation of a mineralized band covering the damaged pulp aligns well with the existing literature. Previous studies have similarly described these radiopaque barriers as effective biological seals that isolate the exposure site from the underlying healthy pulp tissue [28] [29]. The specific morphological variant we observed—characterized by a remarkably large, faint radiopaque mass (Figure 5)—represents a unique healing pattern that was previously described by Linu et al. [29], in 1 out of 13 cases treated with MTA and 3 out of 13 cases treated with BD after an 18‐month follow‐up. This presentation is somewhat analogous to pulpal calcification frequently observed following dental trauma, which is driven by a pulpal cellular response. It can be clinically detected as early as 3 months posttrauma and is characterized by the deposition of hard tissue within the pulp space and subsequent crown discoloration. However, the atypical phenotype we observed, characterized by a distant dentin bridge with a connecting radiopaque branch (Figure 6), has not been previously detailed in the endodontic literature [30]. This finding highlights the clinical value of high‐resolution 3D imaging in uncovering the diverse and complex spectrum of pulpal regenerative responses that conventional 2D radiography might obscure.

### 4.1. Limitation of the Study

Several constraints should be considered when interpreting the present findings. First, an important biological limitation stems from the demographic and baseline pulpal status of the selected sample. The study restricted participants to a narrow age range of 18–25 years as this demographic most frequently requires the extraction of structurally intact premolars for orthodontic purposes. While this ensured a completely healthy, uninflamed pulpal baseline prior to mechanical exposure, it inherently reduced biological variability. Because young adults typically possess a more robust pulpal healing capacity and enhanced cellular regenerative potential, our findings might overestimate the clinical success rates compared to those of older populations. Furthermore, this orthodontic extraction model and its pristine physiological condition contrast with typical clinical scenarios where DPC is primarily performed on teeth affected by deep caries or trauma. In such real‐world cases, preexisting inflammation levels inherently differ, which can significantly alter pulpal healing dynamics, thereby limiting the direct generalizability of our results to broader routine clinical practice.

Second, regarding the study timeline, because extraction dates depended on individualized orthodontic schedules, follow‐up intervals were grouped into predefined time windows (9–12 and 13–16 weeks) rather than exact fixed days. Furthermore, this relatively short follow‐up period, capped at a maximum of 16 weeks, is adequate for capturing early‐stage mineralization but possesses inherent observational constraints. Specifically, it does not allow us to assess long‐term pulp vitality, the complete maturation of the dentin bridge, or the potential incidence of late clinical failures. Therefore, definitive conclusions regarding the long‐term clinical stability of these capping agents over a multiyear period cannot be drawn.

Third, while the split‐mouth design effectively controls for interindividual variability, the overall cohort of 44 teeth (11 teeth per subgroup) remains relatively small. Although key findings remained robust after applying the strict Bonferroni correction for multiple testing, this modest sample size inherently limits the broader generalizability of the study. Furthermore, it increases the risk of Type II errors for secondary parameters and constrains the statistical power required to detect more subtle morphological or clinical differences between the two calcium silicate materials. Consequently, these results should be corroborated and expanded upon by further large‐scale, longitudinal randomized clinical trials.

Fourth, regarding blinding, while the radiologist performed the measurements independently, absolute blinding was challenging because MTA and BD exhibited different radiopacities on CBCT scans.

Lastly, while CBCT provides valuable 3D assessments of the macroscopic morphology, its application presents inherent limitations. Clinically, higher radiation exposure and cost restrict its routine use for DPC follow‐up compared to conventional radiography. Furthermore, noninvasive imaging cannot confirm the true microscopic quality of the dentin bridge, functional tubular structures, or pulpal inflammatory cell presence. To address this, we are currently conducting follow‐up histological investigations to elucidate these parameters in future publications.

## 5. Conclusion

In conclusion, both MTA and BD are highly effective clinical agents for DPC, successfully maintaining clinical pulp vitality over the 9–16‐week observation period. However, BD demonstrated significantly superior performance in the quality of mineralized tissue repair, consistently promoting thicker and more continuous dentin bridges than MTA. Therefore, while both calcium silicate materials are clinically reliable for pulp preservation, BD provides a more predictable, rapid, and structurally robust reparative dentin barrier. Furthermore, this study confirms that small‐FOV CBCT is an invaluable and precise diagnostic tool for the early 3D evaluation of reparative dentin morphology.

**Nomenclature**
MTA: Mineral trioxide aggregate
BD: Biodentine
DPC: Direct pulp capping
ICC: Intraclass correlation
CBCT: Cone‐beam computed tomography
3D CMOS: 3D complementary metal‐oxide‐semiconductor
FOV: Field of view.

## Author Contributions

**Huynh-Anh Bui**: conceptualization, methodology, investigation, data curation, software, writing – original draft, writing – review and editing. **Van-Khoa Pham**: formal analysis, validation, writing – review and editing.

## Funding

This research was funded by the University of Medicine and Pharmacy at Ho Chi Minh City (Grant 263/2024/HĐ‐ĐHYD, dated August 27, 2024).

## Disclosure

All authors have read and approved the final version of the manuscript. Van‐Khoa Pham had full access to all of the data in this study and takes complete responsibility for the integrity of the data and the accuracy of the data analysis.

## Ethics Statement

This study was reviewed and approved by the Ethics Council in Biomedical Research of the University of Medicine and Pharmacy at Ho Chi Minh City, Viet Nam (Approval Number 305/ĐHYD‐HĐĐĐ, 21/05/2019). All procedures performed in studies involving human participants were in accordance with the ethical standards of the institutional and/or national research committee and with the 1964 Helsinki Declaration and its later amendments or comparable ethical standards. The informed consent was obtained from parents of all the participants or participant herself, and all methods were performed in accordance with the relevant guidelines and regulations.

## Consent

Informed consent had been obtained from all subjects and/or their legal guardian(s) for the publication of identifying information/images in an online open‐access publication.

## Conflicts of Interest

The authors declare no conflicts of interest.

## Data Availability

The authors confirm that the data supporting the findings of this study are available within the article and/or its Supporting Information.
